# Direct Powder Extrusion 3D Printing of Praziquantel to Overcome Neglected Disease Formulation Challenges in Paediatric Populations

**DOI:** 10.3390/pharmaceutics13081114

**Published:** 2021-07-21

**Authors:** Janine Boniatti, Patricija Januskaite, Laís B. da Fonseca, Alessandra L. Viçosa, Fábio C. Amendoeira, Catherine Tuleu, Abdul W. Basit, Alvaro Goyanes, Maria-Inês Ré

**Affiliations:** 1 IMT Mines Albi-Carmaux, CNRS UMR 5302, Centre RAPSODEE, Campus Jarlard, Université de Toulouse, CEDEX 09, 81013 Albi, France; mariare@mines-albi.fr; 2 Oswaldo Cruz Foundation, Rua Sizenando Nabuco, 100 CEP 22.775-903, Rio de Janeiro 21040-900, Brazil; lfonseca@far.fiocruz.br (L.B.d.F.); alessandra.vicosa@far.fiocruz.br (A.L.V.); fabio.amendoeira@incqs.fiocruz.br (F.C.A.); 3 Department of Pharmaceutics, UCL School of Pharmacy, University College London, 29-39 Brunswick Square, London WC1N 1AX, UK; patricija.januskaite.18@ucl.ac.uk (P.J.); c.tuleu@ucl.ac.uk (C.T.); 4 FabRx Ltd., 3 Romney Road, Ashford, Kent TN24 0RW, UK; 5 I+D Farma Group (GI-1645), Departamento de Farmacología, Farmacia y Tecnología Farmaceutica, Universidade de Santiago de Compostela, 15782 Santiago de Compostela, Spain

**Keywords:** 3D printing, 3D printed drug products, printing pharmaceuticals and medicines, personalized therapeutics, oral drug delivery systems and technologies, taste masking, translational pharmaceutics, material extrusion additive manufacturing, M3DIMAKER printer, pediatric treatments

## Abstract

For the last 40 years, praziquantel has been the standard treatment for schistosomiasis, a neglected parasitic disease affecting more than 250 million people worldwide. However, there is no suitable paediatric formulation on the market, leading to off-label use and the splitting of commercial tablets for adults. In this study, we use a recently available technology, direct powder extrusion (DPE) three-dimensional printing (3DP), to prepare paediatric Printlets™ (3D printed tablets) of amorphous solid dispersions of praziquantel with Kollidon^®^ VA 64 and surfactants (Span™ 20 or Kolliphor^®^ SLS). Printlets were successfully printed from both pellets and powders obtained from extrudates by hot melt extrusion (HME). In vitro dissolution studies showed a greater than four-fold increase in praziquantel release, due to the formation of amorphous solid dispersions. In vitro palatability data indicated that the printlets were in the range of praziquantel tolerability, highlighting the taste masking capabilities of this technology without the need for additional taste masking excipients. This work has demonstrated the possibility of 3D printing tablets using pellets or powder forms obtained by HME, avoiding the use of filaments in fused deposition modelling 3DP. Moreover, the main formulation hurdles of praziquantel, such as low drug solubility, inadequate taste, and high and variable dose requirements, can be overcome using this technology.

## 1. Introduction

Schistosomiasis is considered one of the most prominent neglected diseases in public health, affecting around 250 million people in more than 70 countries worldwide [1,2,3,4]. Even more alarming is the number of school-aged children (5–14 years old) requiring treatment, which has been estimated to be around 25 million [2,5,6]. The disorder, caused by parasitic worms, is responsible for the highest morbidity and mortality rates in developing countries [7]. To this day, approximately 90% of cases are found in Africa and South America, specifically Brazil, reaching 8–10 million cases of *Schistosoma mansoni* [2,7,8].

Praziquantel (PZQ) has been the standard treatment for over 40 years, and is included on the WHO Model List of Essential Drugs [9,10]. It is also used in preventive chemotherapy due to its efficiency, low cost, and promising safety results [10,11]. Although school-aged children have been the primary target to treat the disease, together with toddlers and children under six years of age, there is currently no suitable formulation available for paediatric patients on the market [3,4].

The current treatment for children is based on the off-label use of PZQ, and the dose adjustment is carried out by splitting commercial tablets for adults [3,4,12]. However, such an approach is dangerous, as dosing errors lead to potential toxic side effects or lack of treatment effect [9]. In conjunction, PZQ has an unpleasant taste and the splitting of tablets favours taste bud exposure to the bitter drug, leading to the rejection of the medication and, consequently, to therapeutic ineffectiveness [9]. Palatability is a determining factor in dosage form acceptability and patient adherence to treatment, especially for paediatric oral formulations [13].

The development of a suitable PZQ formulation for children is challenging since: (a) it exhibits a low water solubility (0.02 mg/mL—class II of the Biopharmaceutics Classification System (BCS)) [14], (b) a variable dose dependent on the patient’s weight is needed to achieve a standard 40 mg/kg dose to treat preschool-aged children [3], and (c) it requires the use of an effective taste masking technology.

Various approaches have been evaluated to overcome the aforementioned obstacles, one being the transformation of the drug solid state structure from crystalline to amorphous [15,16,17,18]. The use of an amorphous form of the drug requires its physical stabilisation in the formulation, which can be achieved by the formation of amorphous solid dispersions (ASDs). The principle of an ASD is based on the dispersion of drug in an amorphous carrier, generally a polymer, generating a homogeneous mixture of reduced molecular mobility [19]. In addition to solubility enhancement properties, the dispersion of the drug within the polymer matrix could be expected to contribute towards taste masking. Solid dispersions of paracetamol and Eudragit^®^ E have already been studied and demonstrated successful taste masking of the drug [20,21]. The combination of both properties (solubility improvement and taste masking) is an important requirement for paediatric patients. This patient group presents a great diversity in terms of anatomy and physiological and psychological responses when compared to adults, and as a result, varied therapeutic responses. Although the palatability of a drug is not the only factor that affects the acceptance of a drug, for unpalatable drugs, it is commonly listed as the first cause of non-adherence to treatment, especially in children [13,22,23].

ASDs can be prepared using different technologies, one being hot melt extrusion (HME), a process in which a material is melted or softened under an elevated temperature and pressure, and is forced through a die by rotating screws [24]. HME has been recently used to obtain filaments for the preparation of personalised medicines using three-dimensional (3D) printing [25]. Three-dimensional printing (3DP) is an innovative additive manufacturing technique, capable of converting 3D computer models into real objects by the sequential deposition of material in a layer by layer manner [26,27,28,29,30,31,32,33,34,35,36]. Currently, the most evaluated 3DP technique in the pharmaceutical area is fused deposition modelling (FDM), as a result of the low printer cost, the good quality of the final product, and the direct use of filaments obtained by HME [37]. Many HME–FDM studies have shown the potential opportunities of preparing medicines with different drugs [38], designs [39], and release profiles [35,40,41], even for paediatric formulations [42].

One of the technical limitations of the FDM 3DP process is the high dependency on the physical and mechanical properties of the filaments for printing feasibility [26,43], and the difficulty of filament preparation [44], especially when high drug loads are required. Recently, direct powder extrusion (DPE), a new 3DP technology that does not require the preparation of filaments using HME and allows the direct extrusion of drug and excipient mixtures in the powder form, was reported [26,45]. This technique has allowed the production of 35 wt% itraconazole tablets via a single step process, with improved solubility characteristics through itraconazole amorphisation during printing [26].

The aim of this study was to use an innovative technology, DPE 3DP, to overcome the main challenges of formulating PZQ for paediatric patients: low drug solubility, unacceptable taste, and requirement for a range of relatively high drug doses, by preparing PZQ ASDs as paediatric Printlets™ (3D printed tablets). The suitability of different powdered materials to directly feed the 3D printer was investigated. The powdered materials tested were physical mixtures of crystalline drug and polymer, and for the first time, pellets and powder forms obtained from ASD–HME extrudates. The characteristics of the resulting printlets were evaluated, with special focus on drug dissolution profiles, taste masking effectiveness, and physical stability.

## 2. Materials and Methods

### 2.1. Materials

Racemic praziquantel (MW 312.4 g/mol) (PZQ) was kindly provided by Farmanginhos/Fiocruz from Brazil. Kollidon^®^ VA 64 (MW 45,000–70,000 g/mol) (KOL) and Kolliphor^®^ SLS Fine (SLS) were donated by BASF Chemical Company, Ludwigshafen, Germany, and Span™ 20 (Span) by Croda International, Snaith, UK. Acetonitrile HPLC/Spectro and Methyl Alcohol HPLC/Spectro came from Tedia Company, Fairfield, USA. For the analysis, distilled and purified water (conductivity of 18.2 MΩ.cm at 23 °C) was obtained by the purification system Milli-Q (classic Purelab DI, MK2) (Elga, High Wycombe, UK). Sodium chloride and potassium phosphate monobasic were obtained from Merck (Darmstadt, Germany), praziquantel reference standard was purchased from USP (Rockville, MD, USA) and sodium phosphate dibasic from Sigma-Aldrich (St. Louis, MO, USA).

### 2.2. Methods

#### 2.2.1. Preparation of the Powdered Materials

Powdered materials with different compositions were tested in the DPE 3D printer (Table 1): physical mixtures (PM) of crystalline PZQ and polymer, pellets (P) produced in a twin-screw extruder by HME and powder obtained by milling the pellets (M).

The physical mixtures (PM 50, PM 35, and PM 35 SLS) were prepared in a Turbula^®^ T2F mixer (96 rpm, 8 min). HME extrudates were prepared using a Thermo-Fisher pharma 16 Extruder (Thermo scientific™, Karlsruhe, Germany), in a co-rotating twin-screw configuration with eight heating zones, two mixing zones, and a screw diameter (D) of 16 mm and L/D ratio equal to 40 (L being the length of the barrel). The heating zones of the extrusion were determined for each of the formulations according to their specific characteristics, ranging from 50 to 180 °C. The HME extrudates were cut into pellets of 1 mm in length. Part of the binary formulation was used as pellets (P 50), and part was milled (M 50). The two ternary formulations (M 35 Span, M 35 SLS) were milled. The milling process was carried out using a Quadro Comil H5 High Energy Mill (Fitzpatrick^®^, Waterloo, ON, Canada) with a mill speed of 8000 rpm and a 610 µm size sieve. The samples were protected from light and kept in a desiccator for conservation.

The three formulations of PZQ processed by HME contained 35 or 50 wt% PZQ, in the presence/absence of surfactants. The addition of the surfactants (Span or SLS) in the formulations was to increase the dissolution of the solid dispersions produced by HME. This investigation was conducted for the formulation containing 35 wt% of PZQ. The surfactant Span (liquid) was added using a peristaltic pump (Thermo Scientific™, Dreieich, Germany) directly into the extruder, while the surfactant SLS (solid) was directly added to the PZQ and polymer during physical mixture preparation. The respective placebo formulations were produced (M Placebo Span and M Placebo SLS) to check the printer’s cleanliness by contaminating the batches with PZQ prior to printing.

#### 2.2.2. DPE 3D Printing

The prepared mixtures, pellets or milled extrudates, were then added to the hopper of a M3DIMAKER™ pharmaceutical 3D printer (FabRx Ltd., London, UK) with a direct powder extruder nozzle as previously reported [26]. AutoCAD 2014 (Autodesk Inc., San Rafael, CA, USA) was used to design the templates of the printlets, which were then exported as a stereolithography (.stl) file into the 3D printer software (Repetier host V 2.1.3, Willich, Germany). The selected 3D geometry was a cylindrical printlet (10 mm diameter × 3.6 mm height). The printer settings in the Repetier Host software were as follows: feed 2100 steps/mm, infill 100%, high resolution with brim, without raft, speed while extruding (20 mm/s), speed while travelling (90 mm/s), number of shells (2), and layer height (0.20 mm). The flow rate, extruder temperature, and feed rate were adjusted for each formulation (Table 1).

**Figure 1:**
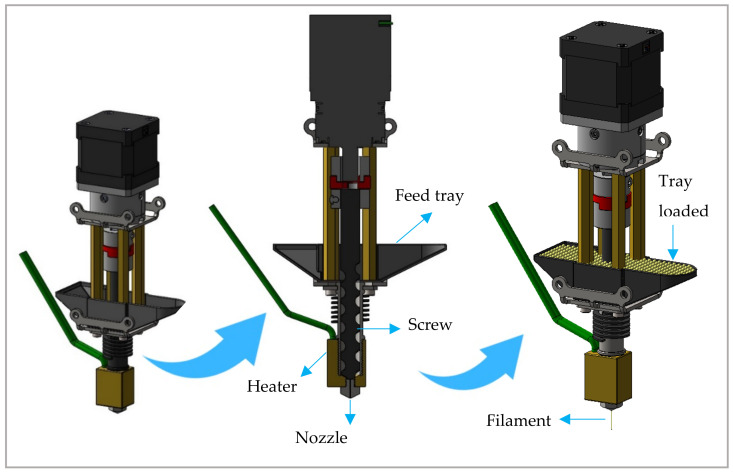
Design of the single-screw direct powder extruder FabRx 3D printer, M3DIMAKER™.

The 3D printer used (FabRx Ltd., Kent, UK) was specifically designed with a direct single-screw powder extruder and a nozzle diameter of 0.8 mm. Its design is based on a single-screw HME with rotation speed (and hence extrusion) controlled by the 3D printer software (Repetier-Host V 2.1.3, Willich, Germany). Furthermore, the extruder nozzle moves in three dimensions to create the objects in a layer-by-layer fashion (Figure 1).

After printing each formulation, the extruder was removed from the printing platform and the screw and barrel were dismounted and washed to avoid cross-contamination between different formulations.

#### 2.2.3. Printlet Characterisation

##### Dimensions

The physical dimensions of the printlets were measured using a digital Vernier calliper and a Sartorius Entris 124-1S analytical balance to determine the mass of each printlet.

##### Optical Microscopy

Images were obtained using a Leica EZ4 HD^®^ microscope (Leica, Wetzlar, Germany) with an integrated high-definition digital camera, set to 8× magnification. The images were then edited with the Leica LAS EZ software program. For calibration purposes, an image (obtained under the same conditions) of a standard slide containing a straight 1 cm segment, with 100 divisions, was employed.

##### Scanning Electronic Microscopy (SEM)

SEM printlet images were obtained using a scanning electron microscope HITACHI TM3030 Plus (Hitachi, Tokyo, Japan) with an acceleration voltage of 15 kV. Samples were fixed on a support using a double-sided adhesive and covered with platinum using a high-resolution SEM coated spray Polaron SC7640 (Quorom Technologies, Lewes, UK).

##### X-ray Powder Diffraction (XRPD) Analysis

Discs of 20 mm diameter × 2 mm height from the same printlet formulations were 3D printed and analysed. A Philips X’Pert Panalytical X-ray diffractometer (Malvern Panalytical, Malvern, UK) using CuKα radiation, 40 mA of current, and 45 kV of voltage was used. The recording spectral range was set at 7–50° with a measuring step (angular deviation between 2 consecutive points) of 0.0167° and an acquisition time of 100 s per point. In addition, each disc was rotated in its sample holder (1 revolution/s) during the results acquisition. Disc printing was performed only for feeding with HME-extrudate powder. X-ray powder diffraction (XRPD) analysis was performed for the ground raw and extruded materials using the same method.

##### Differential Scanning Calorimetry (DSC)

The analysis was performed using a DSC Q200 with the base module and modulated DSC (mDSC) (TA instruments, New Castle, DE, USA). An RCS90 cooling system was used to precisely control the cooling rate. Nitrogen (N_2_) was used as the purging gas at 50 mL/min, and the analysis was carried out in non-hermetic aluminium pans. Indium standards were used for enthalpy and temperature calibration, and an empty aluminium pan was used as a blank control. As for mDSC, sapphire was used to calibrate the instrument for specific heat capacity (Cp) measurements. Samples were heated at a rate of 2 °C/min from 10 to 180 °C, with a modulation period of 40 s and an amplitude of 0.2 °C. Two samples of each printlet (average weight of each sample: 2–6 mg) were analysed: one taken from the border and the other taken from the core.

##### Raman Microscopy

Raman mapping was performed at room temperature (25 °C) using a Raman 300R Alpha confocal microscope (WITec GmbH, Ulm, Germany), equipped with a laser at a wavelength of 532 nm. Samples were analysed by a 50× objective on the surface and deep mapping (8 × 8 μm KOL, 5 × 5 μm PZQ, and 10 × 10 μm and 10 × 20 μm P and M printlets, respectively) was applied to predict drug and polymer distributions.

##### Determination of Drug Loading

One printlet of each formulation was placed in a volumetric flask with 60:40 acetonitrile: water mixture (50 mL) under ultrasound for 5 min until complete dissolution (*n* = 2). Samples of the solutions were then filtered through a 0.22 μm PTFE filter (Millipore Ltd., Dublin, Ireland) and the concentration of drug was determined by external standardisation. Quantification was carried out using a fresh standard stock solution prepared each time before starting the analysis. The standard solution of PZQ was prepared by dissolving 9 mg of PZQ in mobile phase to obtain a final concentration of 0.18 mg/mL. Each sample solution was prepared and analysed in duplicate. The results were expressed as a % of PZQ recovery.

PZQ was determined in the printlets by a high-performance liquid chromatography (HPLC) system (Shimadzu Scientific Instruments, Kyoto, Japan) comprising a diode array UV detector (SPD-10A VP), a pump (LC-10AD VP), an autosampler (SIL-20A VP), and an interface (SCL-10A VP) for the acquisition of data through a software (Ez Start).

The validated HPLC assay involved the injection of 10 µL samples through a Protosil C18 column at room temperature (25 °C) (150 × 4.60 mm–5 µm) (Phenomenex, Bologna, Italy) in an isocratic mode with mobile phase consisting of methanol and water (60:40 *v*/*v*) at a flow rate of 1.5 mL/min. A wavelength of 210 nm was used for detection. The retention time of PZQ was found to be approximately 3 min and the run time was set at 10 min.

##### In Vitro Drug Release Studies

The drug release profiles of the printlets were monitored using a USP-II paddle apparatus (DT 60) (ERWEKA, Heusenstamm, Germany). Paddle stirrers at a speed of 50 rpm and temperature of 37 ± 0.5 °C were used in each test. The printlets were placed at the bottom of a 900 mL vessel of 0.1M HCl media (without surfactant). During the dissolution test, 5 mL samples were taken and filtered through 0.22 µm PTFE filters, and the drug concentration was determined by HPLC (method described in Section 2.2.3 (Determination of Drug Loading)). Tests were conducted in triplicate. Data are reported throughout as mean ± standard deviation.

##### Assessment of Taste Masking Efficiency Using a Novel Biorelevant Buccal Dissolution Test

The in vitro method described by Keeley et al. [46] was used to predict the taste of PZQ released from the milled extrudates and printlets in a simulated buccal environment.

The simulated salivary fluid (SSF) (Sodium chloride—8 g/L; Potassium phosphate monobasic—0.19 g/L; Sodium phosphate dibasic—2.38 g/L; pH 7.4) [47] kept under magnetic stirring at 37 °C ± 1 °C, was pumped through the ‘buccal dissolution column’ using a peristaltic pump at a rate of 1 mL/min, corresponding to an average adult normal total simulated saliva flow range. The other two adjacent parts were composed of wire mesh discs, placed either side of the column. After inserting the sample in the centre of the column lumen, from the top, aliquots were collected at 60, 120, 180, 240, 300, 360, 420, 480, 540, and 600 s, filtered through a 0.22 um membrane, and PZQ analysed by HPLC. Tests were conducted in triplicate. Data are reported throughout as mean ± standard deviation using Microsoft Excel (Microsoft Corp., Redmond, WA, USA) software (version 2016 MSO).

The PZQ taste-concentration profile was previously determined with the rat brief-access taste aversion (BATA) model [48]. It was found that the half maximal inhibitory concentration (IC50) was 0.06 mg/mL, and the taste threshold was 0.03 mg/mL. The classification proposed by Mohamed-Ahmed et al. [48] was used to classify levels of PZQ released at different time points as fully tolerated, well tolerated, tolerated, aversive/untolerated, or highly aversive/highly untolerated, and predict taste masking efficiency of the different formulations.

##### Stability Study

The samples were stored in a Memmert HPP260eco climatic chamber (Memmert, Schwabach, Germany) at 25 °C and 60% relative humidity (RH) for a period of 3 months. The printlets were packaged in amber glass bottles, while the printed discs were stored in transparent glass bottles protected by aluminium. All bottles were closed with plastic screw caps. They were monitored by DSC (printlets) and XRPD (discs) analysis over the period of storage.

## 3. Results

### 3.1. Physical Printlet Characteristics

**Figure 2:**
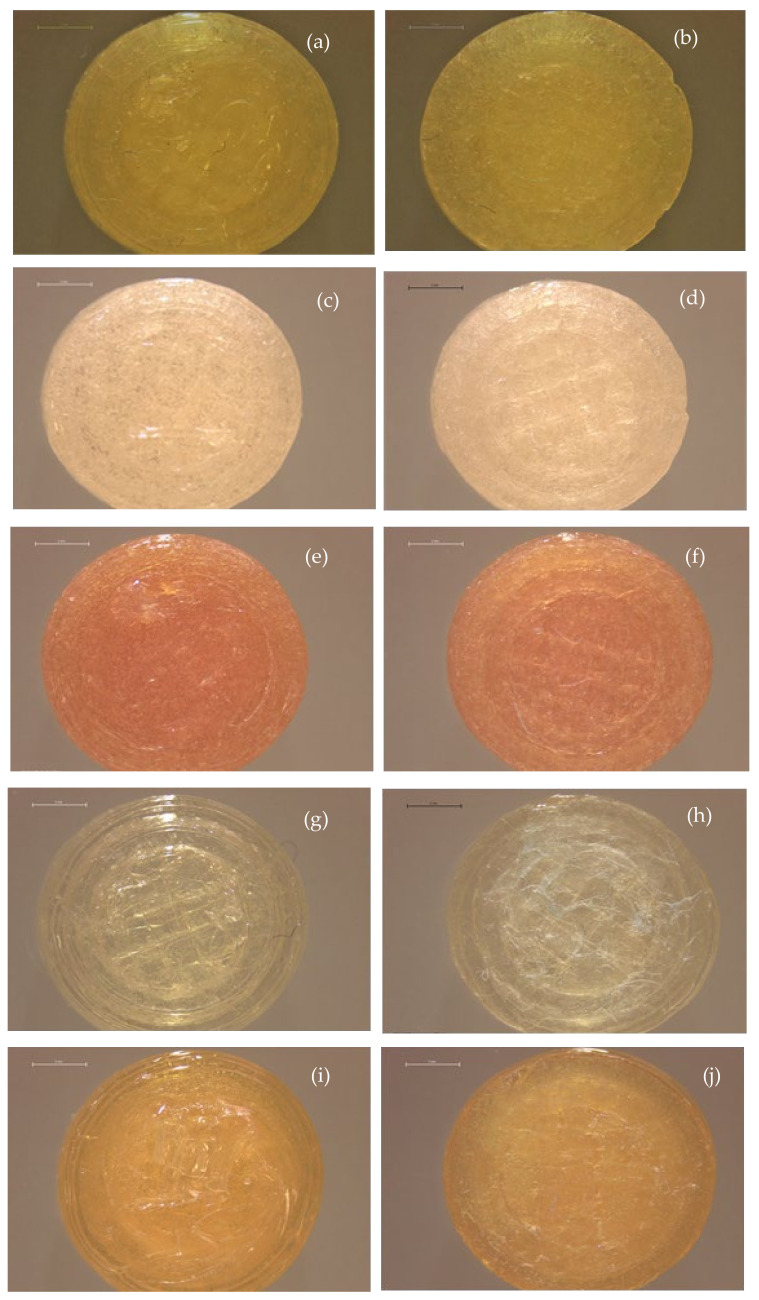
Top (**left** images) and bottom (**right** images) image of printlets obtained with: (**a**,**b**) M 50, (**c**,**d**) M 35 Span, (**e**,**f**) M 35 SLS, (**g**,**h**) M Placebo Span and, (**i**,**j**) M Placebo SLS. Scale refers to 2 mm.

PZQ printlets with different compositions were investigated in the present work to find the best solution for a PZQ formulation to treat schistosomiasis in children. The work was designed to obtain PZQ printlets and to demonstrate the feasibility of producing medications with personalised doses in printlets of 10 mm diameter and 3.6 mm height. The pictures of the best formulations are shown in Figure 2.

The physical mixture of PZQ and KOL (PM 50) was directly added to the hopper of the 3D printer extruder, but the feeding material hampered the printing process and led to unsatisfactory final products (pictures not shown). Varying mass feed rates and screw speed parameters were tested, but none produced printlets with a satisfactory visual quality or enough extruded material. The poor flow of the mixture and electrostatic forces caused high variation in the feeding during printing, making it impossible to produce a continuous and homogeneous flow of material through the screw.

A similar and unsatisfactory behaviour was verified when another physical mixture of PZQ (PM 35 SLS) was directly added to the hopper of the 3D printer extruder. These results showed that, for 3DP with direct powdered material feeding, powder characteristics capable of providing fluidity and homogeneity are essential to a regular flow through the extruder [49]. Without adding additional excipients to improve the flow properties of physical mixtures, it was not possible to obtain good quality printlets from both PZQ formulations.

For the third tested formulation (PM 35 Span), printing was not possible since the mixture could not be prepared with the same conditions used for HME extrusion (addition of the liquid surfactant directly into the extruder).

Alternative processed materials were directly fed into the 3D printer: pellets obtained by HME, and powders obtained from the milled pellets. The main objective of printing using pellets was to facilitate the overall process and overcome the need for milling. Although it was possible to print with pellets, the flow into the printer was inconsistent, most likely due to the size (~1 mm) and the lack of homogeneity in the materials’ particulate morphology. The printing of the printlets using the sample P 50 was possible, however, for a limited number of printlets, since the cleaning of the screw was necessary after each unit produced. For this reason, only DSC analysis was performed for this sample to verify the thermal behaviour after printing and during the stability testing period.

In contrast, milled materials provided a continuous flow, and the printing process was notably improved. However, it was evidenced that the feed rate was impacted by the drug load in the system. The samples in the milled form with a load of 35 wt% PZQ (M 35) showed greater ease of continuous printing compared to the sample containing 50 wt% PZQ (M 50). As a result, even the M 50 formulation presented limitations in the printlet reproducibility. Due to this, SEM and palatability analyses were not performed. Even with these limitations, the characterisations present in this work are an important step as they demonstrate the possibility of printing with high drug load materials obtained by HME.

The images obtained by optical microscopy of PZQ printlets are shown in Figure 2. The variation in printlet colour is related to the differing composition of each one. After the HME process, both tested formulations containing surfactant (M 35 Span and M 35 SLS) had a yellow appearance, with the most intense coloration for the one containing SLS. This is most likely because the surfactant interacts with other formulation components (polymer and drug), resulting in a colour change. This feature was constant for the respective printlets produced with the M 35 Span and M 35 SLS formulations. Interactions (chemical and/or physical) between API and excipients can affect characteristics such as stability, chemical nature, and bioavailability of the API, resulting in efficacy and safety impacts [50,51]. Further studies, such as forced degradation, should be conducted in addition to other spectroscopic techniques (e.g., Fourier-transform infrared spectroscopy, UV-Vis) [52] to better understand the colour differences between the formulations. Another characteristic that can be identified in the optical microscopy images is the greater opacity of the printlet containing 50 wt% PZQ (M 50) compared to the others having only a 35 wt% drug load. When comparing the M 35 Span and M 35 SLS printlets to the respective placebos, the absence of PZQ configures even more transparency. Nonetheless, all printlets presented a good physical appearance, with a smooth surface and shiny finish.

**Figure 3:**
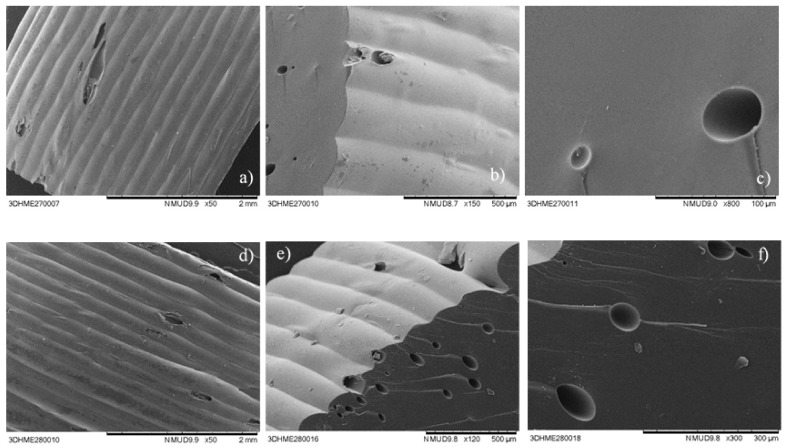
SEM images of printlets: (**a**–**c**) obtained with M 35 Span, and (**d**–**f**) obtained with M 35 SLS.

SEM cross-section images of two PZQ printlets (M 35 Span and M 35 SLS) are shown in Figure 3. They display a dense and homogeneous inner matrix, in which some small holes can be observed. These were most likely formed from air bubbles entrapped during the printing process. The images also provide a clear view of the consistent layers formed by the deposition of material during printing.

The printlet dimensions and weight are reported in Table 2. P 50 and M 50 printlets showed a higher mass variation (0.270 and 0.298 mg, respectively), presumably due to the difference in flow rate (75 and 90%, respectively), as a slower flow rate results in less material deposition. PZQ drug loading was determined only for the M 50 printlets based on the number of units available, and was close to the theoretical load value (48.4%), showing that the dose is approximately 150 mg.

Formulations M 35 with surfactants (Span and SLS) showed a homogeneous flow during the printer feeding process and good uniformity was achieved with regard to physical dimensions (Table 2). PZQ content values of milled extrudates before printing were 34.84% (M 35 Span) and 33.25% (M 35 SLS). After printing, the results remained similar for both formulations (35.02 and 33.54%, respectively), and with that, the dosages of the printlets were found to be around 100 mg. One of the most discussed applications of 3DP for medicine manufacture is the easy adjustment of dose via the manipulation of the size, structure, or shape of the solid dosage form. The adjustment of the dose by changing the size of 3D printed formulations was already demonstrated in the first clinical study of printlets prepared in a hospital for children [53].

As an initial stage in developing a paediatric pharmaceutical formulation, characteristics such as pharmacokinetics and pharmacodynamics, potential routes of administration, toxicity relationship, and taste preferences should be evaluated [54].

In general, it can be argued that from birth to approximately 16/18 years, individuals are considered paediatric patients [55]. However, when analysing this heterogenous age group, it is easy to see significant differences in the physiological development of the body and the need for personalised medicine development for the different stages of childhood [54]. The 3DP approach can therefore produce different designs, resulting in new dosage forms with specific and unique pharmacokinetic characteristics.

Placebo printlets were also analysed for drug content to check for any possible contamination of printer parts. For all placebo printlets, the presence of PZQ was not identified, thus indicating that cleaning the printer between each batch was effective, with no drug contamination throughout.

### 3.2. Physicochemical Characterisations

**Figure 4:**
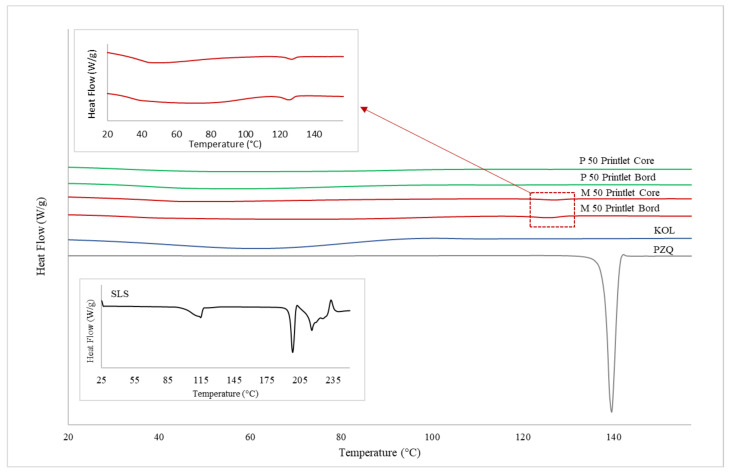
DSC thermograms for P 50 and M 50 printlets, performed on two different parts of each unit (core and border).

DSC, XRPD, and Raman mapping were used to characterise the solid state of the printlets. DSC thermograms confirmed the crystalline nature of the pure drug by the presence of a sharp melting endotherm at ~140 °C, and the amorphous state of the polymer KOL (Figure 4), as reported previously in the literature [56,57,58,59]. Although an endothermic event was observed in the HME powdered material and mainly after milling (data not shown), its low intensity allows us to conclude that P 50 and M 50 powdered materials were predominantly amorphous before printing due to the melting process in HME. These physical characteristics were maintained after printing for both formulations, where the P 50 remained amorphous while the DSC thermogram of the M 50 printlet shows an endothermic peak visualised in the range of 120–122 °C with ΔHfusion 2.15 J/g. However, the crystallinity is similar to that evidenced in the material before printing and cannot be attributed to the presence of the crystalline PZQ ($T_{m}$ ~ 136 °C) or one of the polymorphs of PZQ, B, or C, for which the melting temperatures described in the literature are different (around 106 and 112 °C, respectively) [58,59,60]. The endothermic event shown in Figure 4 can be related to the effect of the polymer on lowering the melting temperature of the drug in the mixture. Figure 4 also shows the homogeneity of the printlets, with no differences in their characteristics measured in the core and on the border.

Although M 50 printlets presented a mixture of crystalline and amorphous material, it was identified that over three months of storage under controlled conditions (25 °C, 60% RH), the endothermic signal and the enthalpy of fusion detected after production remained unchanged (data not shown), evidencing no change in the solid state exposed to the storage conditions.

**Figure 5:**
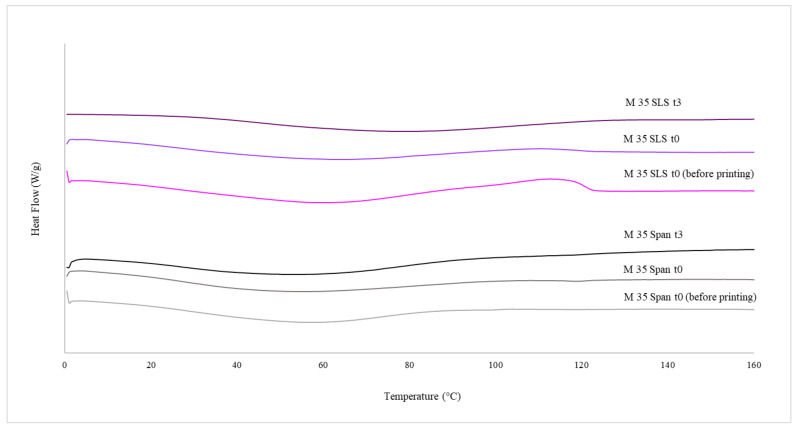
DSC thermograms for M 35 Span and M 35 SLS powdered materials (before printing), and their respective printlets immediately after printing (t0) and after three months of storage (t3).

Figure 5 displays the DSC thermograms for printlets containing 35 wt% PZQ with surfactants (Span or SLS). The amorphous nature of M 35 Span and M 35 SLS materials subjected to HME before printing was confirmed, with no evidence of crystalline PZQ due to the absence of a melting event and the presence of a single glass transition ($T_{g\;M\;35\;SPAN}\;$33 °C and $\Delta C_{p}\;$0.37 J/g.; $T_{g\;M\;35\;SLS}\;$25 °C and $\Delta C_{p}\;$0.36 J/g., respectively). The amorphous pattern after printing (M 35 Span and M 35 SLS printlets) remained unchanged (t0), and no evolution was detected by DSC analysis after three months of storage at 25 °C, 60% RH (t3).

**Figure 6:**
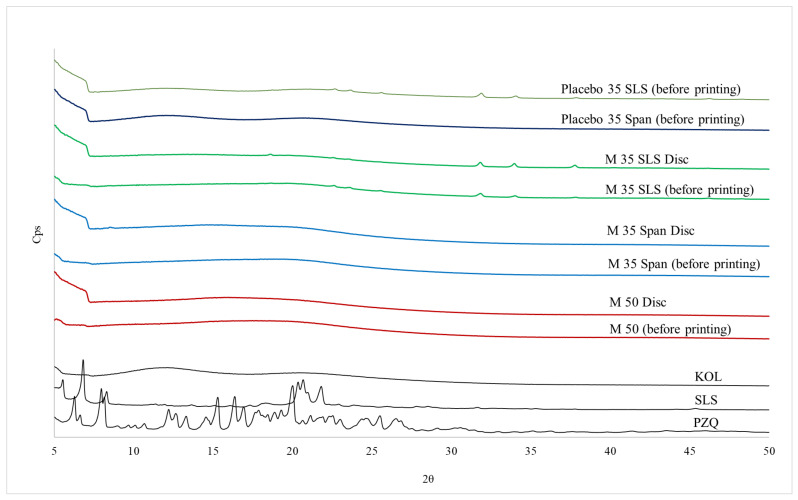
XRPD diffractograms for raw materials (PZQ, SLS, and KOL), ASDs (M 50, M 35 Span, and M 35 SLS), and their respective 3D printed discs.

XRPD diffractograms of all raw, powdered, and printed materials are grouped in Figure 6. The crystalline nature of the raw PZQ and SLS are confirmed by the sharp peaks, in line with the data already described in the literature for the commercial racemic molecule [14,60]. Likewise, the polymer (KOL) also presents a characteristic halo of amorphous material. The amorphous nature of powdered materials and their respective printlets (M 35 Span) previously found in the DSC analysis are also confirmed by XRPD diffractograms. However, the M 35 SLS printlet was the only material presenting some diffraction signals, indicating the presence of crystalline material. It could be verified during this study that these signals are also identified in absence of the drug in the placebo formulation (Figure 6), being related to some structural change in the surfactant (SLS), which was not further investigated here. Unlike the DSC results, the printlet M 50 showed no signs of diffraction, indicating that the sample may be amorphous or the drug in crystalline state is in a proportion under the detection limits of the XRPD technology.

**Figure 7:**
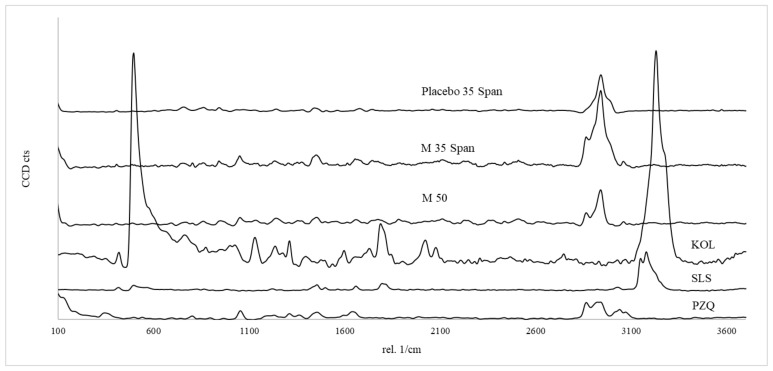
Raman microscopy results for the raw materials (PZQ, SLS, and KOL) and printlets (M 50, M 35 Span, and Placebo 35 Span).

Raman microscopy was performed to provide information about the distribution of PZQ in the printlets. Figure 7 shows Raman spectra for PZQ and KOL and their respective characteristic signals (1054, 1451, 2866, 3061 cm^−1^, and 749, 862, 1740, 2940 cm^−1^, respectively). The results showed the presence of PZQ in the M 35 SLS and M 50 printlets (2879, 2873, 3068, and 3071 cm^−1^), but it is not possible to detect Raman characteristic peaks of the surfactant (SLS), most likely due to the small proportion used (5 wt%) (Figure 7).

**Figure 8:**
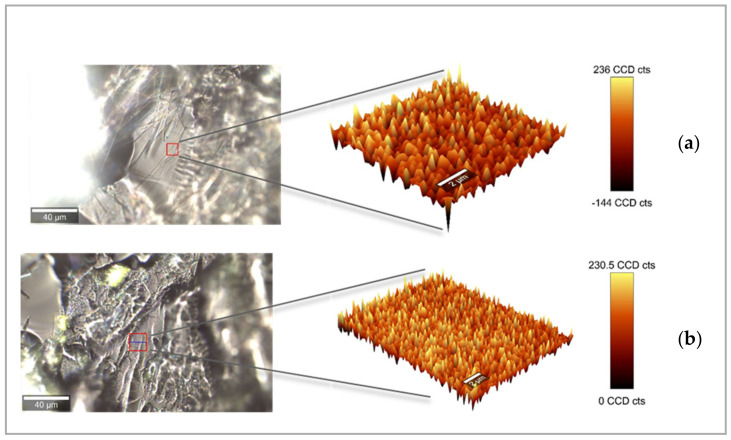
Raman 3D mapping for: (**a**) M 50 printlet with an area of 10 × 10 microns, and (**b**) M 35 Span printlet with an area of 15 × 15 microns.

3D images obtained from the integration of PZQ and KOL peaks revealed each material’s distribution in the analysis area (Figure 8a,b). It is possible to visualise the graphs with different colour intensities that reflect the distribution of PZQ in the printlet. Areas that appear light yellow indicate high absorbance (high concentration of drug), and areas with a darker colour (black) indicate a lower concentration of drug present due to the low absorbance [61]. Thus, these colour variations can map the homogeneity and concentration of the dispersed drug on the screened printlet area. The even distribution of yellow and orange peaks confirms the good distribution level of the PZQ within the polymer matrix, a critical quality attribute for the physical stability of amorphous solid dispersions.

For the M 35 SLS printlet, several analyses were performed, with two different lasers (532 and 785 nm), different integration times, and different laser intensities. In all these analyses, the fluorescence phenomena remained important for the perimeter of Raman images. Therefore, it was impossible to obtain a spectrum on this sample, certainly due to the darker printlet colour.

### 3.3. Dissolution Profiles

**Figure 9:**
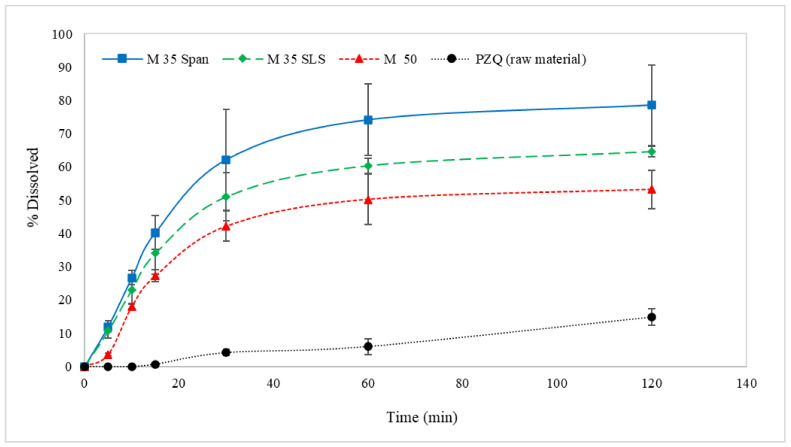
In vitro dissolution profiles of PZQ (raw material) and printlets (M 50, M 35 SLS, and M 35 Span).

The dissolution profiles of M 50, M 35 Span, and M 35 SLS printlets show a greater than four-fold increase in drug release after 2 h compared to PZQ alone (Figure 9). It is important to mention that all printlets were printed directly with powdered materials obtained from HME extrudates without the addition of other excipients that could improve properties such as flowability, dissolution, disintegration, and taste masking. This proves that 3DP can be a highly valuable technology for producing personalised pharmaceutical dosage forms to improve physical and sensory properties necessary for a large variety of medicines. The combination of HME and 3DP techniques could be useful to overcome challenges in the formulation development of BCS class II drugs with low solubility, which represent more than 70% of new drug candidates in the pipeline [62].

It is known from the drug-polymer composition-temperature previously determined by the authors (data not shown) that, at room temperature, 35 wt% PZQ in KOL corresponds to a supersaturated amorphous binary system. Considering that the highest concentration of PZQ (50 wt%) would be sensitive to recrystallisation, in the present study, M 35 Span and M 35 SLS printlets were chosen to be evaluated for taste masking performance using an in vitro biorelevant buccal dissolution method described previously by Keeley et al. [46].

**Figure 10:**
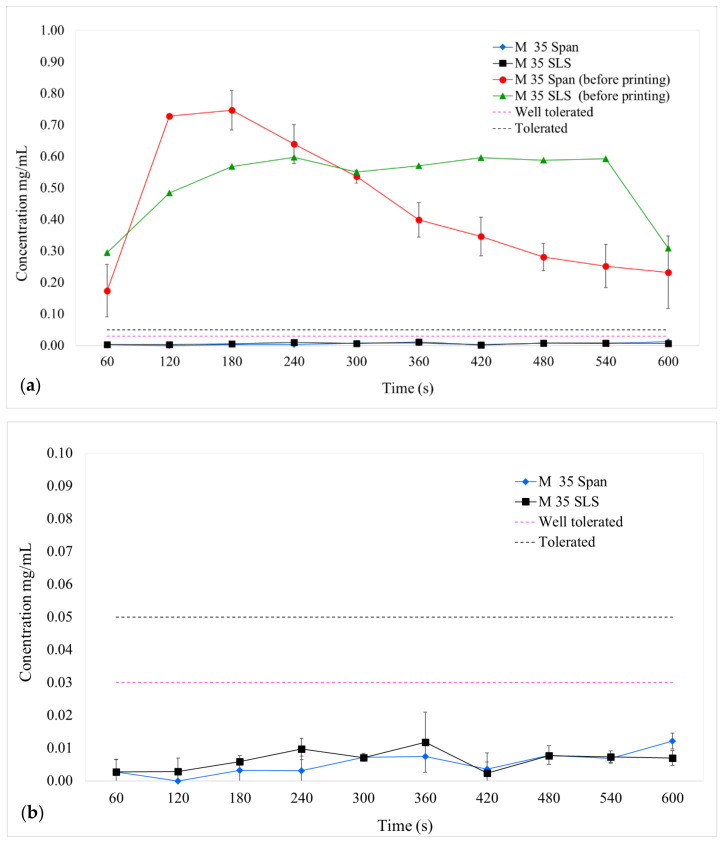
(**a**) PZQ release (mean ± SD) for M 35 Span and M 35 SLS materials (before printing) and their respective printlets, and (**b**) detail of the 3D printed formulation PZQ release. The taste thresholds from a previous study are represented as pink and purple dashed lines.

M 35 Span and M 35 SLS milled materials before printing released more than 0.2 mg/mL of PZQ in the artificial saliva in the first minute of the experiment (Figure 10a). Münster et al. [63] found that the half maximal inhibitory concentration (IC50) for a PZQ taste response was 0.06 mg/mL when tested with the rat BATA model. The dashed lines indicate the PZQ taste tolerability thresholds they found and are classified as tolerable (0.05 mg/mL) and well tolerable (0.03 mg/mL). Therefore, when ingesting a drug in the particulate form, i.e., a powder formulation for dispersion, the bitter and unpleasant taste of PZQ is likely to be present in the mouth immediately upon administration, at a level triggering aversion. The 3D printed formulations exhibited a significantly lower drug release, even after 600 s, than the M 35 Span and M 35 SLS HME milled extrudates before printing (Figure 10a), demonstrating that the 3DP step was key for successful taste masking. As shown in Figure 10b, both printlets (M 35 Span and M 35 SLS) were below the threshold of good tolerability of the PZQ and even further below the PZQ IC50 (0.06 mg/mL), indicating that the formulations provided efficient taste masking without the use of additional taste masking excipients.

The bespoke flowthrough oral dissolution apparatus was used in a previous study to evaluate chlorphenamine maleate, a bitter BCS class I drug, incorporated in sugar spheres and coated with different technologies and polymer coatings [46]. In this study, the system could discriminate the taste masking capabilities of the formulations, using taste thresholds generated with the rat BATA model and confirmed with human taste thresholds.

In the case of PZQ, this in vitro taste assessment is a very useful tool to screen formulations and evaluate printlets in early-stage formulation development. The method is simple to execute, relatively inexpensive, and can guide development decisions so that animal experimentation can be reduced as only one taste profiling of the drug is needed. However, although the preliminary results of the printlets are encouraging, there is still the possibility of improving the final formulation with additional excipients to further favour taste masking.

## 4. Conclusions

For the first time, 3D printed tablets containing 35 and 50 wt% praziquantel (PZQ) were successfully produced by direct powder extrusion (DPE) 3DP from HME extrudates (in pellet and powder form), reducing the dependence on the strict mechanical properties of HME filaments for FDM 3DP.

Printlets with adequate dimensional characteristics and two different doses (~150 and 100 mg) were produced. In vitro dissolution studies showed a greater than four-fold increase in PZQ from the printlets when compared to pure PZQ, most likely due to the predominantly amorphous solid state of PZQ after printing. In addition to the improved performance in the dissolution studies, in vitro taste masking results revealed that the formulations would be within acceptable taste thresholds (tolerated and well tolerated), with enhanced features for paediatric patients.

Most of the paediatric users of PZQ are found in developing countries, with a large variation in the children’s weight due to the poor dietary conditions, showing a wide range of physiological and pharmacokinetic characteristics within a heterogeneous age group. Due to the absence of an appropriate paediatric formulation, treatment of schistosomiasis patients involves the breaking or crushing of a standard adult tablet to achieve the required dose of 40 mg/kg [64,65,66].

The printlets in this work were developed with a focus on paediatric patients, and although the drug loading still requires optimisation, promising results with amorphous systems have been obtained in taste masking and possible dose reduction compared to what is currently available [63,67]. The results from this work demonstrate the unique potential of 3DP in customising PZQ medicines from amorphous systems, specifically for paediatric patients.

## Figures and Tables

**Table 1:** Printlet compositions and DPE printing parameters.

Formulation Code	Composition (wt%)				Printing Parameters		
	PZQ	KOL	SLS	Span	Printing Temperature (°C)	Flow Rate (%)	Feed Rate (%)
**Physical mixtures (PM)**							
PM 50	50	50	-	-	145–170	100–140	100
PM 35	35	65	-	-	145–170	100–140	100
PM 35 SLS	35	60	5	-	140–200	100–140	100
**Pellets (P) and milled powder (M) obtained from HME extrudates produced in a twin-screw extruder**							
P 50	50	50	-	-	140	90	100
M 50	50	50	-	-	135	75	100
M 35 Span	35	60	-	5	130	75	100
M 35 SLS	35	60	5	-	130	75	100

**Table 2:** Printlet characteristics.

Measured Characteristics of Printlets			
Printlet Formulation	Weight (g)	Diameter (mm)	Height (mm)
P 50 *	0.270 ± 0.02	9.720 ± 0.22	3.579 ± 0.05
M 50 *	0.298 ± 0.01	9.810 ± 0.16	3.554 ± 0.14
M 35 Span **	0.297 ± 0.02	9.982 ± 0.25	3.507 ± 0.09
M 35 SLS **	0.290 ± 0.04	9.841 ± 0.22	3.591 ± 0.11

* Values represent the standard deviation, *n* = 8 and ** *n* = 18.
